# Post mortem cerebrospinal fluid α-synuclein levels are raised in multiple system atrophy and distinguish this from the other α-synucleinopathies, Parkinson's disease and Dementia with Lewy bodies

**DOI:** 10.1016/j.nbd.2011.08.003

**Published:** 2012-01

**Authors:** P.G. Foulds, O. Yokota, A. Thurston, Y. Davidson, Z. Ahmed, J. Holton, J.C. Thompson, H. Akiyama, T. Arai, M. Hasegawa, A. Gerhard, D. Allsop, D.M.A. Mann

**Affiliations:** a Division of Biomedical and Life Sciences, Faculty of Health and Medicine, University of Lancaster, Lancaster, LA1 4AY, UK; b Neurodegeneration and Mental Health Research Group, School of Community Based Medicine, University of Manchester, Hope Hospital, Salford, M6 8HD, UK; c Department of Neuropsychiatry, Okayama University Graduate School of Medicine, Dentistry and Pharmaceutical Sciences, 2-5-1 Shikata-cho, Okayama, 700–8558, Japan; d Department of Molecular Neuroscience, Institute of Neurology, University College London, Queen Square, WC1N 3BG, London; e Cerebral Function Unit, Salford Royal Hospitals NHS Foundation Trust, Hope Hospital, Stott Lane, Salford, M6 8HD, UK; f Department of Psychogeriatrics, Tokyo Institute of Psychiatry, 2-1-8 Kamikitazawa, Setagaya-ku, Tokyo, 156–8585, Japan; g Department of Molecular Neurobiology, Tokyo Institute of Psychiatry, 2-1-8 Kamikitazawa, Setagaya-ku, Tokyo, 156–8585, Japan

**Keywords:** Parkinson's disease, Dementia with Lewy Bodies, Multiple system atrophy, Alpha synuclein, Cerebrospinal fluid

## Abstract

Differentiating clinically between Parkinson's disease (PD) and the atypical parkinsonian syndromes of Progressive supranuclear palsy (PSP), corticobasal syndrome (CBS) and multiple system atrophy (MSA) is challenging but crucial for patient management and recruitment into clinical trials. Because PD (and the related disorder Dementia with Lewy bodies (DLB)) and MSA are characterised by the deposition of aggregated forms of α-synuclein protein (α-syn) in the brain, whereas CBS and PSP are tauopathies, we have developed immunoassays to detect levels of total and oligomeric forms of α-syn, and phosphorylated and phosphorylated oligomeric forms of α-syn, within body fluids, in an attempt to find a biomarker that will differentiate between these disorders. Levels of these 4 different forms of α-syn were measured in post mortem samples of ventricular cerebrospinal fluid (CSF) obtained from 76 patients with PD, DLB, PSP or MSA, and in 20 healthy controls. Mean CSF levels of total and oligomeric α-syn, and phosphorylated α-syn, did not vary significantly between the diagnostic groups, whereas mean CSF levels of phosphorylated oligomeric α-syn did differ significantly (p < 0.001) amongst the different diagnostic groups. Although all 4 measures of α-syn were higher in patients with MSA compared to all other diagnostic groups, these were only significantly raised (p < 0.001) in MSA compared to all other diagnostic groups, for phosphorylated oligomeric forms of α-syn. This suggests that this particular assay may have utility in differentiating MSA from control subject and patients with other α-synucleinopathies. However, it does not appear to be of help in distinguishing patients with PD and DLB from those with PSP or from control subjects. Western blots show that the principal form of α-syn within CSF is phosphorylated, and the finding that the phosphorylated oligomeric α-syn immunoassay appears to be the most informative of the 4 assays would be consistent with this observation.

## Introduction

Idiopathic Parkinson's disease (PD) is one of several neurodegenerative disorders that can present with similar clinical symptoms, particularly parkinsonism which is a combination of tremor, rigidity and bradykinesia. Progressive supranuclear palsy (PSP), corticobasal syndrome (CBS) and multiple system atrophy (MSA) are neurodegenerative conditions that are neuropathologically distinct entities, but show clinical overlap with PD. Because of the prominent clinical features they show in addition to parkinsonism, they are often described as “atypical” Parkinsonian syndromes.

In vivo diagnosis of PD and atypical Parkinsonian disorders relies on clinical criteria (Poewe and Wenning, 2002). Although none of these disorders is currently curable, it is important to make the correct diagnosis as early as possible since the symptomatic therapeutic approaches differ, and future (causative) therapies might be targeted directly against the underlying pathological process in each of these disorders. Reliable, early clinical diagnosis is also crucial for correct classification of patients within clinical trials (Schlossmacher and Mollenhauer, 2010). Nonetheless, clinical diagnosis of PD is often imprecise, particularly during the early stages of the illness. Indeed, clinicopathological studies have shown that only 69–70% of people with autopsy-confirmed PD had, in life, at least two of the cardinal clinical signs of the disease, and 20–25% of people with two of these symptoms had a pathological diagnosis other than PD (Hughes et al., 1992, 2001). There is clearly an urgent need to develop a biomarker for PD and the related disorder of Dementia with Lewy bodies (DLB) which will not only distinguish these disorders from normal people, but also from patients with other parkinsonian and/or dementing syndromes. Considerable effort therefore currently goes into the development of biomarkers for PD and the atypical parkinsonian disorders that would reliably allow the clinician to distinguish between them at an early stage.

PD and DLB are both characterised pathologically by the deposition of aggregated forms of α-synuclein protein (α-syn) in the brain in the form of neuronal cytoplasmic inclusions (Lewy bodies, LBs) and dystrophic processes (Lewy neurites, LNs) (Spillantini et al., 1997, 1998). In PD, α-syn pathology is principally found in brain stem and mid brain structures (substantia nigra, locus caeruleus, dorsal motor vagus, and nucleus of Meynert) (Spillantini et al., 1997), whereas in DLB the similar α-syn changes are focussed on regions such as cingulate cortex, parahippocampal gyrus and amygdala (Spillantini et al., 1998). LBs and LNs contain a misfolded, fibrillar and phosphorylated form of α-syn (Anderson et al., 2006; Spillantini et al., 1997). In demented PD patients (PDD), there is a ‘spread’ of α-syn pathology into cortical structures, and PD, PDD and DLB may form a continuum of disease. Pathological changes also involving α-syn, but chiefly in glial cells, characterise MSA. Collectively, PD, DLB and MSA are often referred to as ‘α-syncleinopathies’ (Spillantini et al., 1998). PSP and CBS on the other hand are tauopathies.

We, and others, have previously reported that α-syn can be detected within cerebrospinal fluid (CSF) and plasma (El-Agnaf et al., 2003, 2006; Tokuda et al., 2006, 2010). This extracellular form of α-syn seems to be secreted from neuronal cells by exocytosis (Emmanouilidou et al., 2010; Lee et al., 2005) and could play an important role in cell-to-cell transfer of α-syn pathology in the brain (Angot and Brundin, 2009). Consequently, levels of α-syn within plasma and/or CSF might therefore serve as a biomarker for PD, and other α-synucleinopathies (i.e. DLB, MSA). Here, we have tested whether ventricular post mortem CSF measures of α-syn can predict the presence or amount of α-syn pathology within the brain in α-synucleinopathies, and can differentiate the α-synucleinopathies from each other, as well as from other parkinsonian disorders, such as progressive supranuclear palsy (PSP), which are characterised by tauopathy. Moreover, because pathological investigations have demonstrated that the aggregated α-syn within LBs and LNs is phosphorylated (at Ser 129) (Anderson et al., 2006; Fujiwara et al., 2002; Obi et al., 2008), we have argued (Foulds et al., 2010) that these modified, pathological forms of the protein ought to more accurately reflect the fundamental neuropathology of PD, and that measurements of phosphorylated α-syn within CSF might provide a more direct marker of α-syn pathology in the brain (akin to measurement of tau phosphorylated at Ser 181 (ptau-181) as an index of neurofibrillary pathology in AD (see Blennow and Hampel, 2003 for review)), than the more straightforward measures of ‘total α-syn’ which most previous assays (for example, El-Agnaf et al., 2003, 2006; Tokuda et al., 2006, 2010) have been limited to.

## Materials and methods

All CSF samples and brain tissues had been collected with full Ethical permission, following donation by next of kin, and were kindly provided by the Parkinson's Disease UK Brain Bank (PDUKBB) and Queen Square Brain Bank (QSBB), except for one MSA case from Manchester Brain Bank (MBB). Clinical diagnoses had been made locally by the referring specialist Neurologist in care of the patient. Nonetheless, in all instances, the clinical diagnosis had been confirmed pathologically by Neuropathologists within their respective tissue banks. For PDUKBB cases, clinical information and neuropathological reports were available on PDUK web site. For QSBB and MBB cases relevant information was available locally. All clinical and pathological diagnoses were made in accordance with internationally recognised criteria.

Samples of CSF were obtained at post mortem from 96 individuals (Table 1), 85 were provided by the PDUKBB, 10 by QABB and one from MBB. CSF was drawn directly at post mortem from the subarachnoid space and/or lateral ventricles and immediately frozen and stored at − 80 °C pending analysis. The post mortem delay time between death and obtaining/freezing CSF was variable, ranging from 2 to 96 h, though 62% of samples had been collected within 24 h of death and only 15% after 48 h of death.

Of the 85 samples from PDUKBB, 39 were from patients clinically diagnosed as having PD, 17 patients had DLB, 7 had PSP, 4 had MSA and 18 were controls. Of the 10 samples from QSBB, 5 had PSP, 3 had MSA and 2 were controls. The sample from MBB had MSA. Twenty six of the PD patients were anecdotally reported in their clinical histories as suffering from dementia (PD Dem) and/or cognitive impairment (PD Cog), whereas no evidence of cognitive impairment or dementia had been reported in the other 13 patients who were therefore considered to be cognitively unimpaired (PD nonD). All patients with PSP had classical Steele–Richardson syndrome. All patients with MSA had striatonigral degeneneration (SND) subtype except one with a mixed subtype. Formal neuropsychological testing had not been performed for most of the PD and DLB cases, and MMSE scores were therefore generally not available. One of the QSBB PSP cases scored 23/30 on MMSE and 3 others from PDUKBB were reported as suffering from dementia, but the remaining PSP cases, and all the MSA and control cases had been considered to display no cognitive impairment, or had normal MMSE scores (where available).

Although, overall, age at onset, age at death and duration of illness differed significantly between PD, DLB, PSP, MSA and control groups (F_3,72_ = 2.95, p = 0.039, F_4,95_ = 2.48, p = 0.05, F_3,72_ = 4.55, p = 0.006, respectively) (Table 1), post hoc Tukey test showed no significant differences in any of these measures between any of the diagnostic groups, probably because of the small sample sizes involving PSP and MSA groups, particularly in respect of disease duration (Table 1). There were no significant differences between PD, PDD or DLB groups (F_2,52_ = 0.43, p = 0.654; F_2,53_ = 2.40, p = 0.100; F_2,52_ = 0.845, p = 0.435, respectively).

Paraffin sections (6 μm) of frontal and cingulate cortex, hippocampus and temporal cortex, amygdala and parahippocampus, and substantia nigra were obtained from the PDUKBB and QSBB from the same PD, DLB, PSP and MSA patients, wherever possible. However, sections were only available from 6 of the 20 control subjects (4 from PDUKBB and 2 from QSBB (Table 1).

### Biochemical methods

#### Preparation of recombinant α-syn

Recombinant α-syn (without any purification tag) was prepared at Lancaster University from *E. coli* using the following protocol. pJEK2 was used to transform FB850, a *rec* A^−^ derivative of BL21 (DES) pLysS. FB850 carrying this plasmid was grown in an 800 ml batch culture and protein expression was induced through the addition of isopropyl-β-D-thiogalactopyranoside (IPTG). A protein with a molecular weight of ~ 17 kDa started to accumulate in the cells 30 min after induction and reached maximum levels after 150 min. Immunoblot analysis identified this protein as α-syn using an anti-α-syn mouse monoclonal antibody (MAb 211, from Santa Cruz Biotechnology, Santa Cruz, CA, USA). After a 3 h induction, the suspension was centrifuged, and the cells resuspended in buffer. The cells were lysed by sonication, and then cell debris and insoluble material was removed by centrifugation at 4 °C for 1 h at 30,000 rpm. α-Syn was extracted from the supernatant by ammonium sulphate precipitation, then purified using two chromatography columns; mono Q and Superose 6. After purification, 5 μg of protein ran as a single band when observed on a Coomassie blue-stained SDS gel, corresponding to monomeric α-syn.

#### Preparation of phosphorylated α-syn

Phosphorylated α-syn was prepared from recombinant α-syn as described previously (Sasakawa et al., 2007).

#### Preparation of oligomeric forms of α-syn

To prepare a standard for the oligomeric α-syn immunoassay, the recombinant protein was oligomerised by incubation at 45 μM in phosphate-buffered saline (PBS) in an orbital shaker at 37 °C for 5 days, and the monomer and oligomer were separated by size exclusion chromatography. A sample (0.5 ml) of pre-aggregated α-syn was loaded onto a Superose 6 column (44 × 1 cm) connected to a fast protein liquid chromatography (FPLC) system (Atka Purifier, GE Healthcare) and eluted with running buffer (PBS) at a flow rate of 0.5 ml/min. Absorbance of the eluate was monitored at 280 nm and fractions of 1 ml were collected and protein concentration determined.

To prepare a standard for the phosphorylated oligomeric α-syn immunoassay, the phosphorylated protein was allowed to aggregate by incubation at 50 μM in PBS in an orbital shaker at 37 °C for 5 days. Aggregation of the protein was confirmed by thioflavin T assay (see Supplementary data). In this case, the amount of sample available was too small to fractionate by size-exclusion chromatography.

### Immunoassays

We have already established immunoassays for the measurement of ‘total’ and ‘soluble oligomeric’ forms of α-syn in human biological fluids, including blood plasma and CSF (El-Agnaf et al., 2003, 2006; Tokuda et al., 2006), but these methods have been further optimized here.

### Total α-syn

**Fig. 1:**
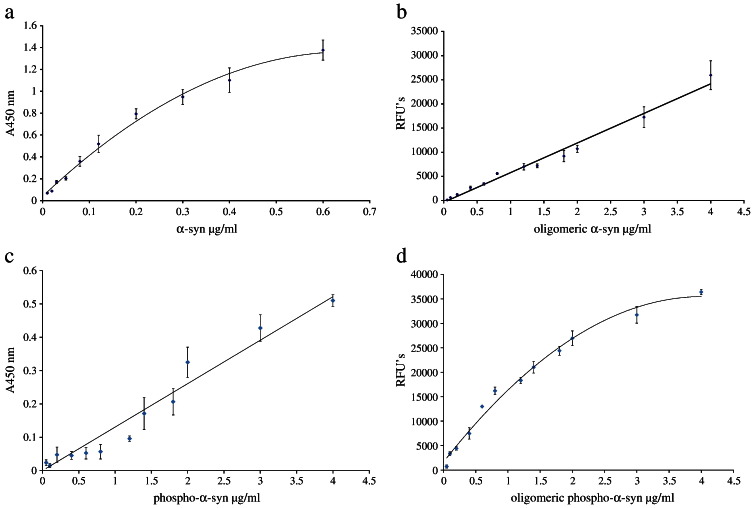
Standard curves for immunoassays. Total α-syn (a), oligomeric α-syn (b), phosphorylated α-syn (c) and oligomeric phosphorylated α-syn (d).

An ELISA plate (Iwaki) was coated with 100 μl/well of anti-α-syn monoclonal antibody 211 diluted 1:1000 (Santa Cruz Biotechnology, Inc., Santa Cruz, CA) (0.2 μg/ml) in 50 mM NaHCO_3,_ pH 9.6, and incubated at 4 °C overnight. The wells were then washed 4 times with PBS containing 0.05% Tween-20 (PBS-T), and incubated for 2 h at 37 °C with 200 μl/well of freshly prepared blocking buffer (2.5% gelatin in PBS-T). The plate was washed again 4 times with PBS-T and 100 μl of the assay standard or CSF samples were added to each well, (each CSF sample was diluted 1:40 with PBS), and the assays were performed in triplicate. Following this, the plate was incubated at 37 °C for 2 h. After a repeat washing with PBS-T, 100 μl/well of the detection antibody, anti-α/β/γ-synuclein FL-140 (Santa Cruz Biotechnology, Inc., Santa Cruz, CA), dilution 1:750 (0.27 μg/ml) in blocking buffer was added, and the plate was incubated at 37 °C for 2 h. After another wash with PBS-T, the plate was incubated with 100 μl/well of secondary antibody (goat anti-rabbit HRP (Sigma), dilution 1:10,000 in blocking buffer at 37 °C for 2 h. The plate was then washed again with PBS-T before adding 100 μl/well of Sure Blue TMB Microwell Peroxidase Substrate (KPL, USA) and leaving the colour to develop for 30 min at room temperature. Finally 100 μl/well of stop solution (0.3 M H_2_SO_4_) was added and absorbance at 450 nm was determined. Recombinant monomeric α-syn was used to create a standard curve (Fig. 1a).

### Oligomeric α-syn

The microtitre plate was coated and blocked using the same method as the assay for ‘total α-syn’. The wells were then washed 4 times with PBS-T and 100 μl of the CSF samples (diluted 1:25 with PBS) or assay standard (oligomeric α-syn) was added to each well, in triplicate. Following this, the plate was incubated at 37 °C for 2 h. After a repeat wash with PBS-T, 100 μl/well of the detection antibody, biotinylated anti-α-synuclein 211 (diluted 1:1000 in blocking buffer) was added, and the plate was incubated at 37 °C for 2 h. After another wash with PBS-T, the plate was incubated with 100 μl/well of streptavidin-europium, diluted 1:500 in streptavidin-europium buffer (Perkin Elmer) and shaken for 10 min. After a further 50 min agitation on a rotating platform, the plate was washed again with PBS-T, before adding 100 μl/well enhancer solution (Perkin Elmer). Finally, the plates were read on a Wallac Victor^2^ 1420 multi-label plate reader, using the time-resolved fluorescence setting for europium.

Oligomeric α-syn was used to create a standard curve (Fig. 1b). The specificity of this assay towards aggregated forms of α-syn was confirmed (the α-syn monomer gave no significant signal).

### Phosphorylated α-syn

The antibody-sandwich immunoassay for ‘total’ α-syn was modified to detect only phosphorylated forms of the protein by replacing the 211 phospho-independent capture antibody with polyclonal anti-α-synuclein N-19 (Santa Cruz Biotechnology, Inc., Santa Cruz, CA, USA), diluted 1:3000 (0.07 μg/ml). The phospho-dependent rabbit monoclonal antibody, Phospho (pS129) Antibody (Epitomics Inc., CA, USA), used at a dilution of 1:3000, was the chosen detection antibody. This antibody only detects α-syn phosphorylated at Ser129. The preferred secondary antibody was human serum absorbed goat anti-rabbit HRP, 1:3000 (KPL, USA, rehydrated in 1 ml H_2_O). This assay did not detect non-phosphorylated recombinant α-syn.

### Oligomeric, phosphorylated α-syn

The antibody-sandwich ELISA for ‘oligomeric’ α-syn was modified to detect only phosphorylated, oligomeric forms of the protein, by replacing the 211 phospho-independent capture antibody with the phospho-dependent rabbit monoclonal antibody, Phospho (pS129) (Epitomics, Inc., CA, USA), used at a dilution of 1:3000. The detection antibody was biotinylated Phospho (pS129) at a dilution of 1:400. Recombinant, oligomerised, phosphorylated α-syn (oligo-pS-α-syn) was used to generate a standard curve (Fig. 1d). This assay did not detect the monomeric form of pS-α-syn.

### Immunoblotting

According to the measures of total and oligomeric α-syn within CSF, cases with relatively high and low concentrations of α-syn were selected for immunoblotting in order to characterise the molecular properties of α-syn within CSF.

For SDS-PAGE, these samples were run on 12.5% polyacrylamide gels and the separated proteins were electrotransferred onto nitrocellulose membranes (0.45 μm, Invitrogen), at 25 V, 125 mA for 75 min, which were then blocked with 5% powdered, skimmed milk dissolved in PBS-T for 1 h. Membranes were incubated overnight with (a) polyclonal antibody, anti-α/β/γ-synuclein (FL-140) (Santa Cruz, USA), dilution 1:3000 or (b) phosphorylated anti-α-synuclein pS129 (Epitomics, USA), dilution 1:3000. The membranes were washed three times in PBS-T, followed by incubation with HRP-conjugated rabbit anti-mouse or goat anti-rabbit (Sigma), as appropriate, at 1:5000 in PBS-T, for 1 h. The protein bands were visualised using ECL reagents (Pierce, Rockford, IL) as described by the manufacturer.

### Haemoglobin assays

Because previous studies have suggested that contamination of CSF samples by blood, either at lumbar puncture or at post mortem, might contribute through lysed red cells to α-syn measures (Hong et al., 2010), we assayed our CSF samples for haemoglobin levels using an immunoassay. The haemoglobin levels in CSF samples were measured using a Human haemoglobin ELISA Quantitation Kit from Bethyl Lab Inc (Montgomery, TX, USA) according to the manufacturer's instructions.

### Histological methods

Wax sections were immunostained for α-syn pathology using the rabbit polyclonal antibody #1175 with microwave pretreatment in 0.1 M citrate buffer pH 6.0. This antibody recognises both phosphorylated and non-phosphorylated forms of α-syn, but in PD and DLB generates identical patterns of immunostaining as pSer129 — an antibody specific to forms of α-syn phosphorylated at Ser129 (Obi et al., 2008).

The severity of α-syn pathology (ie overall density of Lewy bodies and Lewy neurites) within each brain region was rated on a 4 point scale (0 = absent, 1 = occasional/mild, 2 = common/moderate, 3 = numerous/severe), and a composite score across all 5 regions obtained by summation of individual scores, with a maximum score of 20 possible.

### Statistical analysis

All data analysis was performed using SPSS v 16.0. For normally distributed data, two-sample Student's *t*-test for independent samples or one-way ANOVA were applied in comparing means of CSF α-syn measures between two or more groups, respectively. Alternatively, for non-normally distributed data, Kruskal–Wallis analysis of variance with post hoc Mann Whitney *U* test was used. Similarly, when testing the correlations between CSF α-syn measures and age at onset or death, or duration of illness, or haemoglobin concentration Spearman's first rank correlation or Pearson's correlation tests were used as appropriate. All levels of significance were two-tailed and set at p < 0.05.

## Results

**Fig. 2:**
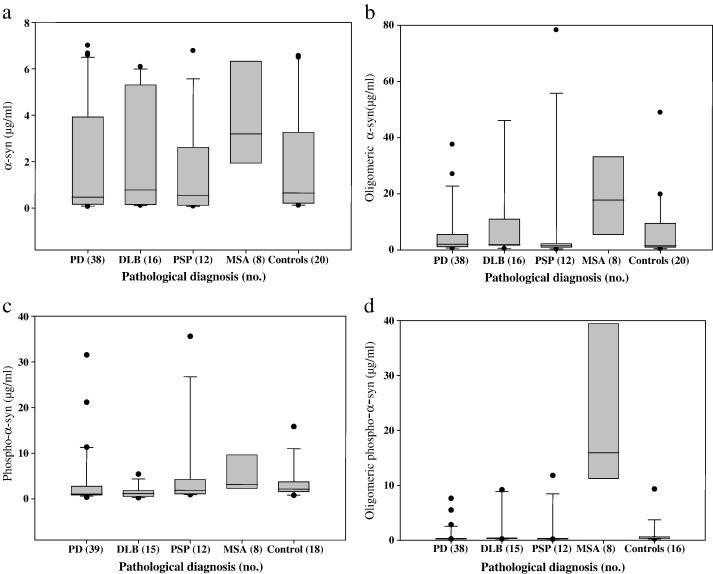
Box-whisker plots for the CSF α-synuclein immunoassay results. The length of each box represents the interquartile range (75–25%) of the sample, the solid line drawn across the box the median, and outliers are denoted by dots. Immunoassay results are presented for each pathological diagnostic group for (a) total α-syn levels, (b) oligomeric α-syn levels, (c) phosphorylated α-syn levels and (d) oligomeric phosphorylated α-syn levels.

There were no significant differences in mean CSF levels of total α-syn (Fig. 2a), oligomeric α-syn (Fig. 2b) or phosphorylated α-syn (Fig. 2c) between PD, DLB, PSP, MSA and control groups (F_4,89_ = 1.36, p = 0.255, F_4,89_ = 1.37, p = 0.249, F_4,87_ = 1.21, p = 0.313, respectively) (Table 2). In contrast, mean CSF levels of oligomeric phosphorylated α-syn (Fig. 2d) were highly significantly different between PD, DLB, PSP, MSA and control groups (F_4,84_ = 22.4 p < 0.001) (Table 2). Post hoc Tukey test showed highly significant differences (p < 0.001) in mean CSF levels of oligomeric phosphorylated α-syn between the MSA and all of the other diagnostic groups (Table 2). There were no significant differences in mean CSF levels of total α-syn, oligomeric α-syn, total phosphorylated α-syn or oligomeric phosphorylated α-syn between PD, PD (Cog) and PD (Dem) groups (F_2,37_ = 1.23, p = 0.303, F_2,37_ = 0.77, p = 0.468, F_2,38_ = 0.53, p = 0.596, F_2,37_ = 1.67, p = 0.202, respectively) although it is noted that the numerical levels of oligomeric phosphorylated α-syn increased progressively from PD through PD (Cog) to PD (Dem) groups (Table 2).

There were no significant correlations between CSF levels of total α-syn, oligomeric α-syn, phosphorylated α-syn or oligomeric phosphorylated α-syn, and pathology scores, expressed either as total pathology score or as severity scores for each individual area, either across all 71 patients or within the PD and DLB patients, separately or combined. Similarly, there were no significant correlations between CSF levels of total α-syn, oligomeric α-syn, phosphorylated α-syn or oligomeric phosphorylated α-syn, with age at onset of disease or duration of illness within PD and DLB patients, either as single or combined groups.

### Immunoblotting

**Fig. 3:**
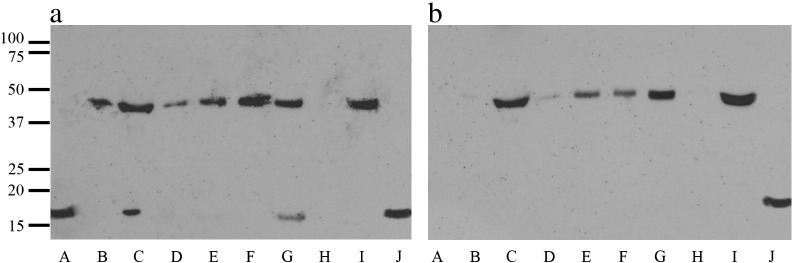
Immunoblots of α-syn (a) and pS-α-syn (b) within CSF of PD, DLB, MSA, PSP and control cases with relatively high and low α-syn immunoassay levels (lanes C, E, G, I and B, D, F and H, respectively) are shown, along with recombinant non-phosphorylated standard (lane A) and recombinant oligomerised, phosphorylated standard (lane J). The immunoblot using polyclonal anti-α/β/γ-synuclein antibody FL-140 (Santa Cruz) (Figure a), shows in most/all samples irrespective of diagnostic status, an α-syn species at ~ 46–48 kDa, which is strongly present in all samples with high CSF α-syn levels (lanes C, E, G and I) but is less strongly present in those with low CSF α-syn levels (lanes B, D, F and H). In two samples with high CSF α-syn levels there is an additional α-syn species at 16 kDa (lanes C and G) which is not present in those with low CSF α-syn levels, but has the same molecular mass as the recombinant protein (lane A). Using the phosphorylated anti-α-synuclein pS129 antibody (Figure b), only the 46–48 kDa species is detected, again this being more strongly present in all samples with high CSF α-syn levels (lanes C, E, G and I) but less strongly present in those with low CSF α-syn levels (lanes). Recombinant α-syn (lane A) is not detected by the phosphorylation specific α-syn antibody (lane A).

Immunoblots of α-syn within CSF, of PD, DLB, MSA, PSP and control cases with relatively high and low absorption α-syn values are shown in Fig. 3. Immunoblots using the polyclonal anti-α/β/γ-synuclein antibody FL-140 (Fig. 3a), showed in most/all samples irrespective of diagnostic status, an immunoreactive band at ~ 46–48 kDa, which was strongly present in all samples with high α-syn CSF levels (lanes C, E, G and I) but was less strongly present in those with low CSF α-syn levels (lanes B, D, F and H). In two samples with high CSF α-syn levels there was an additional α-syn species at 16 kDa which represents the monomeric protein (lanes C and G) and was not present in those with low CSF α-syn levels. Using the phosphorylated anti-α-synuclein pS129 antibody (Fig. 3b), only the 46–48 kDa species was detected, again this being strongly present in all samples with high CSF α-syn levels (lanes C, E, G and I) but less strongly present in those with low CSF α-syn levels (lanes B, D, F and H). Recombinant α-syn at 16 kDa (lane A) was only detected by the FL-140 antibody, and not the phosphorylation dependent α-syn antibody pS129 (see Fig. 3a, lane A). Inspection of the immunoblots (Fig. 3) suggests, therefore, that this 46–48 kDa α-syn species might represent an oligomerised and phosphorylated form of α-syn (the estimated molecular mass would suggest a trimer). Minor quantities of non-phosphorylated (monomeric) α-syn were also present, but these were only detectable in those cases with high CSF α-syn levels.

### Confounding factors

It has been suggested from previous studies (Mollenhauer et al., 2008) that levels of total α-syn within CSF may progressively increase with increasing post mortem delay time. However, we found no correlation between levels of α-syn within CSF and post mortem delay time for any of the 4 immunoassays, either when all 96 patients were considered as a group, or separately according to diagnosis (data not shown). Moreover, mean post mortem delay time did not differ significantly between any of the diagnostic groups (F_4,89_ = 1.90, p = 0.118).

Furthermore, because, some of the CSF samples were obviously contaminated with blood, and because previous studies have suggested that such contamination of CSF samples might contribute to α-syn measures through lysed red cells (Hong et al., 2010), we assayed the CSF samples for haemoglobin. Mean haemoglobin levels within CSF samples did not differ significantly between the various diagnostic groups (PD = 4.6 ± 2.3 μg/ml; DLB = 5.1 ± 2.6 μg/ml; PSP = 5.8 ± 2.2 μg/ml; MSA = 4.4 ± 2.0 μg/ml; Controls = 6.1 ± 3.2 μg/ml; F_4,73_ = 1.01, p = 0.408), nor was there any correlation between CSF haemoglobin level and any one of the 4 measures of α-syn (total α-syn r = 0.168, p = 0.148; oligomeric α-syn r = 0.156, p = 0.178, phosphorylated α-syn r = 0.094 p = 0.418, oligomeric phosphorylated α-syn r = 0.027 p = 0.818).

## Discussion

In the present study, we have shown by immunoblotting that both phosphorylated and non-phosphorylated forms of α-syn can be detected in CSF of patients with PD, DLB, PSP, MSA, and also in control individuals, and that the levels of these can be measured by immunoassay. Consequently, we have not only employed conventional immunoassays to measure total levels of α-syn, as many other groups have done previously, but we have developed new assays based on the detection of phosphorylated and/or oligomerised forms of α-syn, since these may have more relevance in targeting and indexing the pathological species of α-syn that is accumulated in the brains of patients with these disorders (Fujiwara et al., 2002; Anderson et al., 2006; Obi et al., 2008).

The main finding to emerge from this study is the observation that measurement of oligomeric phosphorylated forms of α-syn in CSF can differentiate patients with MSA from all of the other diagnostic groups. Although this finding is based on a relatively small number of MSA cases, and may therefore be considered preliminary, the distinction between MSA and other patient groups was robust, and higher α-syn levels were consistently seen across all 4 assays in MSA compared to PD/DLB and other non-synucleinopathies. Recent findings by Hirohata et al. (2011) showing that an unidentified factor in CSF promotes the *in vitro* aggregation of α-syn, and that CSF from patients with MSA was more effective in this respect than CSF samples from patients with PD, would be consistent with our findings.

There have been a few other recent studies looking at biomarkers in MSA. Aerts et al. (in press) compared total α-syn in lumbar CSF from 47 patients with MSA with those from 58 patients with PD, 3 with DLB, 10 with PSP and others with CBS and vascular parkinsonism, but detected no significant differences in mean level between any of the diagnostic groups. Similarly, Shi et al. (2011) did not find any differences in mean α-syn levels between 32 patients with MSA and 126 with PD, though in both instances such levels were significantly lower than 137 control subjects and 50 patients with Alzheimer's Disease (AD). Mollenhauer et al. (2011) also reported α-syn levels to be decreased in patients with MSA (as well as in those with PD and DLB) compared to patients with AD, and ones with other neurological disorders. As seen in these other studies, we also found that measurements of total α-syn did not clearly distinguish patients with MSA from those with PD (and other parkinsonian disorders).

Our present data therefore suggest that raised levels of phosphorylated forms of α-syn, rather than total α-syn, might provide a test for not only distinguishing MSA from normal individuals, but perhaps more importantly from other synucleinopathies. However, it is accepted that these are very preliminary data and will need verification in larger sample cohorts, especially in samples taken from living patients earlier in the course of their illness before it is possible to categorically state the value of this as a diagnostic test.

There are several potential confounders in our study which we have taken into consideration, leading us to believe that our present findings are genuine.

Firstly, as compared to lumbar puncture where this is less of a problem, it is almost inevitable that the practicalities of taking CSF samples at post mortem will inadvertently include contamination of some samples by red blood cells, which themselves are a potential source of α-syn (Barbour et al., 2008; Hong et al., 2010). Indeed, a recent study (Hong et al., 2010), based on the analysis of CSF samples obtained at lumbar puncture by Luminex assays, noted that levels of α-syn were significantly lower than those of controls, but only when those samples contaminated by blood (with haemoglobin concentrations greater than 200 ng/ml) were excluded from the analysis. Consequently, we assayed our CSF samples for haemoglobin to assess whether varying degrees of blood contamination might have contributed to the output from our immunoassays. Our findings of a lack of correlation between CSF haemoglobin level and any of the α-syn measures, either over all the patients and controls, or within any of the diagnostic groups, makes us confident that the assay results presented here are reliable and have not been influenced to any great extent by contamination by red blood cells. Moreover, it is known that α-syn within platelets is not phosphorylated at serine 129 (Shults et al., 2006), and therefore contamination of CSF samples by blood may not, in any case, be expected to influence the output from the immunoassays for phosphorylated forms of α-syn.

Secondly, it has been suggested from previous studies (Mollenhauer et al., 2008) that levels of total α-syn within CSF may progressively increase with increasing post mortem delay time and hence, the variations in α-syn measured in this study might simply reflect group differences in post mortem delay times. However, we found no correlation between levels of α-syn within CSF and post mortem delay time for any of the 4 immunoassays, either when all 96 patients were considered as a group, or separately according to diagnosis (data not shown). Moreover, mean post mortem delay time did not differ significantly between any of the diagnostic groups. Therefore, we have no reason to believe that the findings presented here are not reliable. To our knowledge, there have been no other studies except that of Mollenhauer et al. (2008) indicating a rise in levels of α-syn within CSF at post mortem, and so it is still uncertain as to whether these levels do indeed change after death.

There have been a number of previous studies investigating whether measurement of total α-syn can also be employed as a biomarker for PD, though these have not always provided consistent results. For example, an earlier study by Tokuda et al. (2006) found levels of total α-syn on average to be lower in samples of CSF taken from a group of patients with PD compared with a group of normal or neurological controls. However, in a later study (Tokuda et al., 2010) no such clear differences were seen. Elsewhere, Mollenhauer et al. (2008, 2011) have similarly reported a lowering of total α-syn levels between PD patients and controls, though this finding has not been replicated by others (Ohrfelt et al., 2009; Spies et al., 2009; Reesink et al., 2010; Aerts et al., in press). In the present study, using a similar immunoassay protocol to Tokuda et al. (2006, 2010), we also found no significant differences in total α-syn levels between PD patients and controls, or between DLB patients and controls. Hence, it remains unclear whether measuring total α-syn levels in CSF has any utility in discriminating between patients with LBD (i.e. PD and DLB) and control subjects.

Because recent studies have suggested that oligomeric forms of α-syn may be the toxic species that induce neuronal cell death, it has been suggested that measurement of these particular forms of α-syn might be better biomarker for PD than total α-syn (Tokuda et al., 2010). Indeed, it has been shown that soluble oligomeric forms of α-syn are elevated in brain homogenates of patients with PD and DLB (Sharon et al., 2003, Paleologou et al., 2009), supporting such an argument. In keeping with this hypothesis, El-Agnaf and colleagues have found elevated levels of oligomeric α-syn in plasma (El-Agnaf et al., 2006) and CSF (Tokuda et al., 2010) of PD patients compared to controls. However, again using similar methodologies, we were unable to replicate these findings with our results showing no clear distinctions between PD or DLB patients and controls with respect to oligomeric forms of α-syn.

It is not clear from a methodological standpoint why we have obtained results dissimilar to those of Tokuda et al. (2010), though it is notable that these latter authors employed CSF samples obtained at lumbar puncture from living patients most of whom were sampled within 5 years of onset of illness. In the present study we employed post mortem samples from end-stage PD and DLB patients dying 11–44 years on average after onset of illness. Hence, increases in oligomeric forms of α-syn early in the course of the disease could diminish with time. This same kind of situation has been seen in Motor Neurone Disease where CSF levels of TDP-43 protein were found to be increased within the first 11 months of illness, but after this time fell and became not significantly different from control subjects (Kasai et al., 2009).

In summary therefore, we present some new methods of assessing α-syn levels in CSF from patients with parkinsonian disorders, and show that those assays based on the detection of phosphorylated oligomeric forms of α-syn may have utility in differentiating patients with MSA from other parkinsonian disorders in which the underlying pathology is also α-syn based (i.e. PD and DLB) or is tau-based (i.e. PSP).

The following are the supplementary materials related to this article.

Supplementary materials.

## Figures and Tables

**Table 1:** Selected clinical and demographic details of cases studied.

Group	Gender	Age at onset (year)	Age at death (year)	Duration (year)
All PD (n = 39)	29M, 13F	64.2 ± 11.8	78.4 ± 6.7	14.2 ± 7.8
PD (n = 13)	10M, 3F	66.1 ± 11.7	79.0 ± 6.5	12.9 ± 6.6
PDD (n = 26)	19M, 10F	63.3 ± 12.0	78.1 ± 6.9	14.8 ± 8.4
DLB (n = 17)	14M, 3F	62.4 ± 8.2	74.0 ± 7.5	11.8 ± 6.9
PSP (n = 12)	10M, 2F	73.5 ± 6.9	80.7 ± 7.9	6.6 ± 3.8
MSA (n = 8)	4M, 4F	64.3 ± 7.6	70.9 ± 7.4	7.6 ± 2.9
Controls (n = 20)	13M, 7F	na	77.9 ± 12.1	na
aControls (n = 6)	5M, 1F	na	73.3 ± 12.4	na

a Those 6 of the 20 control cases for which paraffin sections were available.

**Table 2:** Mean (± SD) CSF levels of α-syn (μg/ml) as determined by each immunoassay in patients with PD (non-demented (nonD), cognitively impaired (Cog) and demented (Dem), DLB, PSP, MSA and normal control individuals.

	Total α-syn (μg/ml)	Oligo α-syn (μg/ml)	pS α-syn (μg/ml)	Oligo pS α-syn (μg/ml)
PD (n = 39)	1.93 ± 2.49	7.04 ± 1.64	3.43 ± 6.18	0.77 ± 1.51
PD (nonD) (n = 13)	1.34 ± 2.16	11.11 ± 2.58	4.41 ± 8.68	0.26 ± 0.03
PD (Cog) (n = 10)	1.47 ± 2.10	2.35 ± 2.02	1.76 ± 1.02	0.68 ± 0.78
PD (Dem) (n = 16)	2.67 ± 2.83	6.37 ± 9.91	3.67 ± 5.73	1.28 ± 2.27
DLB (n = 16)	2.31 ± 2.51	9.47 ± 2.09	1.63 ± 1.42	1.60 ± 3.02
PSP (n = 12)	1.45 ± 1.97	7.91 ± 2.21	5.14 ± 9.73	1.25 ± 3.32
MSA (n = 8)	3.80 ± 2.40	22.49 ± .19	7.14 ± 9.19	19.56 ± 1.66*
Control (n = 20)	1.87 ± 2.29	6.78 ± 1.14	3.58 ± 3.85	1.05 ± 2.23

*Indicates significantly different (P < 0.001) from assay value for patients with PD (overall, and nonD, Cog and Dem subgroups), DLB, PSP and normal control individuals.
