# Supplementary material for: Post mortem cerebrospinal fluid α-synuclein levels are raised in multiple system atrophy and distinguish this from the other α-synucleinopathies, Parkinson's disease and Dementia with Lewy bodies
Source: Neurobiol Dis. 2012 Jan;45(1):188–95. doi: 10.1016/j.nbd.2011.08.003 (PMC3657198; doi:10.1016/j.nbd.2011.08.003)
Supplement: Supplementary file 1 — Supplementary materials. [file mmc1.doc]

*Preparation of the recombinant oligomeric phosphorylated α-synuclein standard*


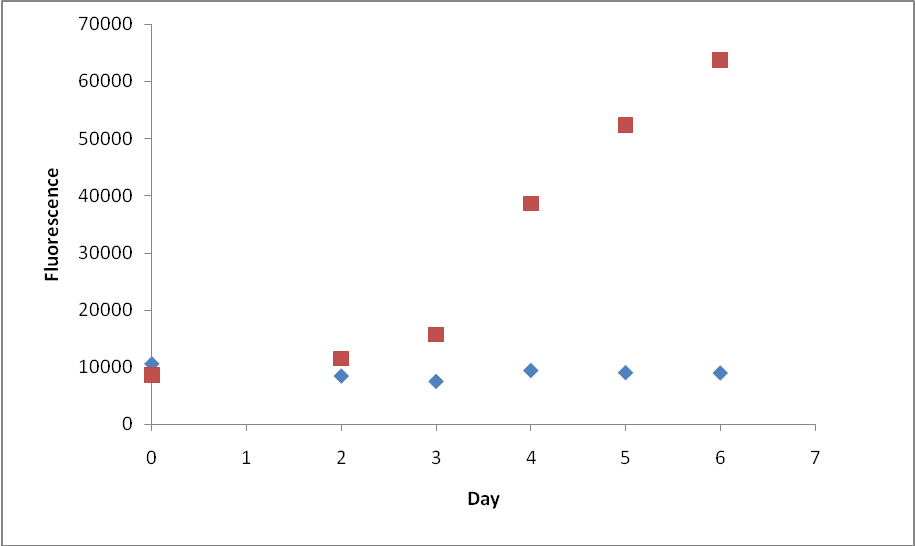


*Supplementary Figure*

The presence of aggregated phosphorylated α-synuclein was monitored by thioflavin T fluorescence. Duplicate 15 μl samples of 24 μM phosphorylated α-syn (in PBS buffer, pH 7.4) were removed after incubation of the protein solution for various periods of time at 37°C. These samples were each added to 15 μl of 40 μM thioflavin T in 50 mM glycine/NaOH (pH 9.0) before the characteristic change in fluorescence was monitored using a BioTek Synergy plate-reader (Exλ = 442 nm and Emλ = 483 nm) (red squares). The plate was read six times, 10 mins apart, immediately after the addition of protein. Controls assays were also performed involving the unaggregated 24 μM phosphorylated α-syn (blue diamonds). Results show the mean value of the samples ± the difference between those mean values. The assay was allowed to run for 6 days to reveal the progression of fibril formation, although the aggregated phosphorylated α-syn sample was removed on day 3.
